# Quantifying the effect email reminders have on patient reported outcome measure returns in a large prostate cancer registry

**DOI:** 10.1186/s41687-022-00426-1

**Published:** 2022-03-07

**Authors:** Nathan Papa, Jonathan G. Bensley, Katrina Hall, Melanie Evans, Jeremy L. Millar

**Affiliations:** Department of Epidemiology and Preventive Medicine, School of Public Health and Preventive Medicine, Monash University, 553 St Kilda Road, Melbourne, VIC 3004 Australia

## Introduction

Systematic collection of patient-reported outcome measures (PROMs) enables researchers to understand trends in clinical care, measure their effects on patient outcomes, and potentially devise interventions to optimise those outcomes [1]. There are impediments to widespread population-based use of PROM surveys. PROM completion is subject to various patient-level factors such as perceived irrelevance of the survey instrument or that inordinate amounts of time required to complete the PROM, difficulty with an electronic mode of completion, and a variety of other phenomena (forgetfulness, differing priorities, procrastination) [2–4]. Therefore, strategies to maximise patient engagement and the completion of PROMs instruments are necessary for collection of robust and unbiased outcomes data [5]. Previous studies have found that reminder emails are effective in increasing the response rate to web-based survey instruments [6, 7]. In a clinical trial setting, automatic reminders appear likely to positively impact completion and timeliness of PROM submission [8].

We sought to quantify the improvement in PROM return rate that a reminder email confers in a large population-based registry setting, without the explicit opt-in consent and active monitoring present in a trial. The Victorian Prostate Cancer Outcomes Registry (PCOR-Vic) is a population-wide clinical quality registry enrolling men with newly diagnosed prostate cancer [9]. As of 2021, coverage is greater than 80% of the incident diagnoses in Victoria, Australia. It operates with an opt-off consent model, i.e., patients are notified that they will be entered into the registry unless they request not to be. PCOR-Vic administers the validated PROM instrument EPIC-26 [10] (Expanded Prostate Cancer Index Composite—26) to patients approximately one year after their initial prostate cancer diagnosis/treatment, predominantly via email contact. Further, we aimed to explore the effect on response rate of patient age at PROM, socioeconomic status, and the day of the week the initial contact email was sent.

## Methods

Data was extracted from the Victorian Prostate Cancer Outcomes Registry (PCOR-Vic), details of which have been previously described [9]. Approximately 12 months after treatment, registry staff make contact via telephone with patients and invite them to answer the EPIC-26 questionnaire. This instrument measures symptoms in the domains of urinary incontinence, irritation, bowel function, sexual function, and vitality. Three methods of completion are offered: by telephone immediately with the staff member, or to be sent the instrument by email or mail for self-completion. The option to complete the EPIC-26 instrument by email was introduced in April 2018, and currently, the majority of patients opt to use this mode. If a response is not received within seven days, an automatic reminder email is then sent the next day. If the patient has still not returned the instrument after a further week, they will be contacted by telephone and reminded to complete the instrument. Responses collected electronically are automatically entered into the registry database.

Patients that elected to receive the EPIC-26 by email from April 2018 to March 2021 inclusive, and have a recorded collection date, formed the analytic sample for this study. The cumulative percentage of electronically returned and answered questionnaires per ordinal day following initial contact was calculated and plotted. Questionnaires returned completely unanswered, or those subsequently completed by telephone or mail, were not counted in the numerator of the cumulative return percentage. A four-parameter logistic curve was fitted to the overall plot to predict the completion rate in the absence of a reminder email. To examine the return rate over time, and the effect of a reminder on this rate, plots reflecting the change in cumulative returned percentage, relative to two days prior, were also generated i.e. [(percentage_day_n_ minus percentage_day_n-2_) / percentage_day_n-2_]. These figures were also generated for the subgroups of patient age, socioeconomic quartile of patient residence, and day of the week of initial email contact. Socioeconomic quartile within the state of Victoria was derived from Australian Bureau of Statistics correspondence to the patient's residential postcode [11]. Further exploration of predictors of electronic completion by day 22 was performed with multivariable logistic regression.

## Results

Within the three-year study period, 5065 patients elected to receive and were sent the EPIC-26 PROM instrument via email (and had a recorded return date); 4673 (92%) returned a completed instrument by email (176 returned the instrument unanswered, 207 completed by telephone, 9 completed by mail), 4456 (88%) within 22 days of initial email contact (Additional file 1: figure S1).

**Fig. 1:**
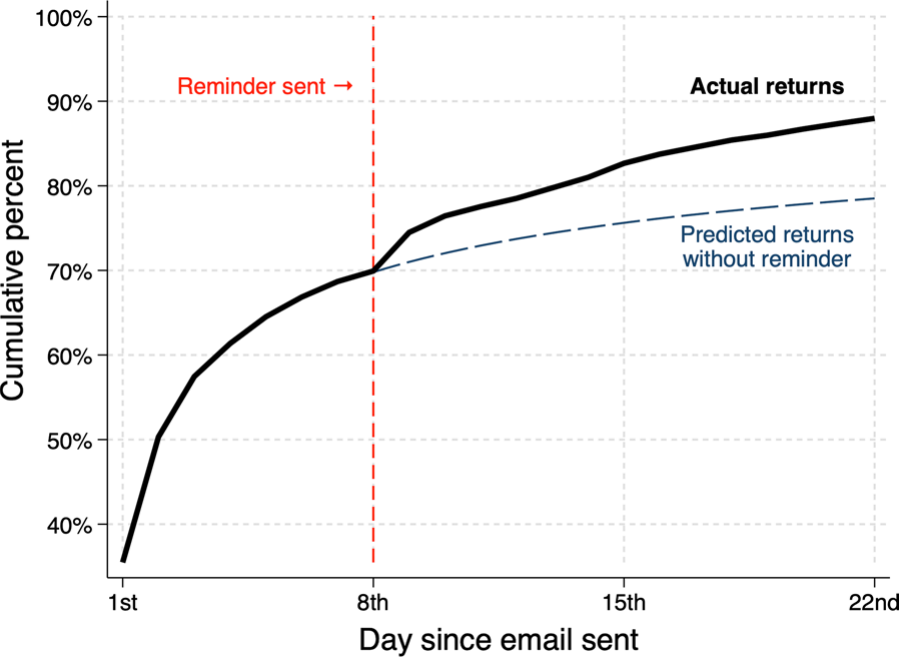
Thick black line = cumulative percentage of EPIC-26 electronic returns by day since email sent. Red dotted line = email reminder sent following 8th day. Blue dotted line = predicted cumulative return percentage without reminder email sent

**Fig. 2:**
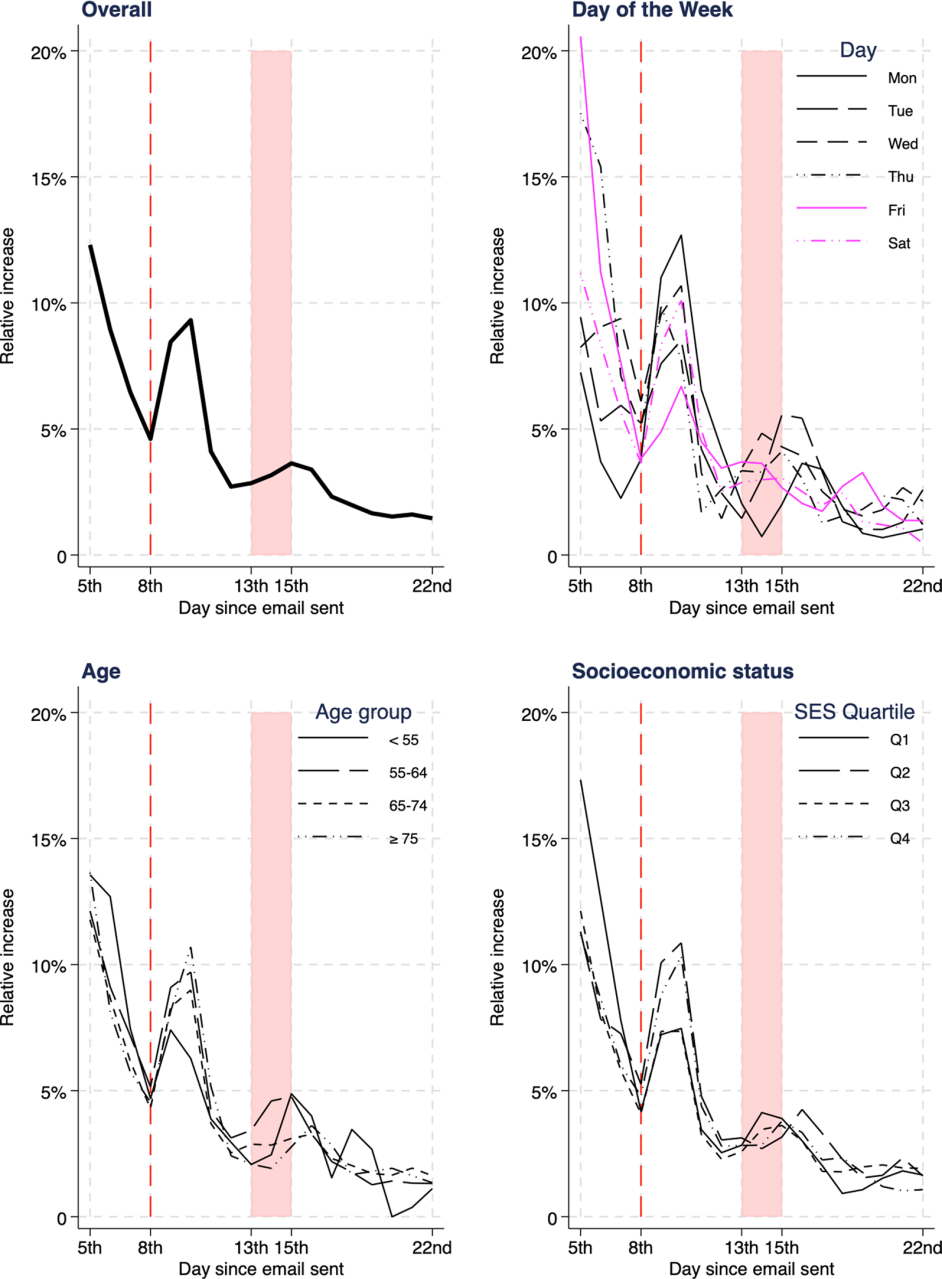
Relative increase of cumulative electronic return percentage compared to two days prior, plotted from 5th day since first email sent. Red dotted line = email reminder sent following 8th day. Red shaded area = time of second phone call reminder from 13 to 15th day since first email sent. Lines plotted for all patients, by day of the week first email sent, age group and socioeconomic quartile

A clear uptick in cumulative returns was noted following the 8th day reminder email (Fig. 1) with the relative increase from the 8th day to the 10th day being 9.3% (Table 1). Utilising the four-parameter logistic regression curve, overall completions at the 22nd day were predicted to be 78.5% without the reminders, 9.5% lower than the absolute proportion observed. Prominent immediate increases in the rate of return after the 8th day were noted for older men aged 75 and over (10.7%) and those who had an initial email sent on a Monday and if required, a reminder scheduled on Tuesday (12.7%) (Fig. 2). Considerably shallower increases were observed in those with an initial email sent on Friday (reminder email scheduled on a Saturday) (6.7%) and men aged under 55 (6.3%), though these younger men appeared more receptive to a phone call reminder at approximately two weeks. No clear association was noted for socioeconomic status.

**Table 1:** Percentage EPIC-26 answered and returned electronically by 8th, 10th and 22nd day since email sent, and relative percentage increase in return from 8 to 10th day

	N (column %)	Cumulative % returned 8th day	Cumulative % returned 10th day	Cumulative % returned 22nd day	Relative percentage increase in return from 8 to 10th day
Overall	5065	69.9	76.4	88	9.3
*Day of the week first email sent*					
Monday	669 (13.2)	69.5	78.3	88.3	12.7
Tuesday	826 (16.3)	68.2	74	86.4	8.5
Wednesday	920 (18.2)	68.2	75.4	88.3	10.7
Thursday	852 (16.8)	71.1	76.6	88	7.8
Friday	848 (16.7)	70.5	75.2	87.4	6.7
Saturday	950 (18.8)	71.9	79.2	89.3	10.1
*Age group*					
< 55	326 (6.4)	68.4	72.7	83.4	6.3
55 – 64	1472 (29.1)	69.3	76	88.6	9.7
65 – 74	2396 (47.3)	71	77.4	88.8	9
≥ 75	871 (17.2)	68.7	76	86.3	10.7
SES quartile*					
1st (most disadvantaged)	774 (15.3)	70.9	76.2	87.3	7.5
2nd	1008 (19.9)	67.6	74.9	87.3	10.9
3rd	1148 (22.7)	71.1	76.3	88.1	7.4
4th (most advantaged)	2123 (42.0)	70.1	77.4	88.5	10.3

*12 missing SES. NB it is an expected result that more incident cases of prostate cancer are from men resident in socioeconomically advantaged areas

Return proportion by the 22nd day was very similar across all subgroups (86.3%—89.3%) with the exception of patients aged under 55 (83.4%) (Additional file 1: Figure S2). In a multivariable model, age category was predictive of response with the 55–64-year-old (OR = 1.58; 95%CI: 1.13 – 2.21) and 65–74-year-old (OR = 1.60; 95%CI: 1.16 – 2.20) categories both more likely to return the questionnaire than the youngest age category (Table 2).

**Table 2:** Predictors of electronically returned questionnaire by 22nd day. OR = odds ratio

	OR (95% CI)
*Day of the week first email sent*	
Monday	1
Tuesday	0.85 (0.63 – 1.17)
Wednesday	1.00 (0.73 – 1.37)
Thursday	0.98 (0.71 – 1.34)
Friday	0.91 (0.73 – 1.25)
Saturday	1.10 (0.81 – 1.51)
*Age group*	
< 55	1
55 – 64	1.58 (1.13 – 2.21)
65 – 74	1.60 (1.16 – 2.20)
≥ 75	1.27 (0.89 – 1.80)
*SES quartile*	
1st (most disadvantaged)	1
2nd	1.00 (0.75 – 1.32)
3rd	1.07 (0.81 – 1.41)
4th (most advantaged)	1.13 (0.88 – 1.45)

## Discussion

PCOR-Victoria is a large registry that covers more than 80% of prostate cancer diagnoses in Victoria, Australia. The opt-off model ensures high population coverage, but also minimises the contact between patients and registry staff who do not have a role in clinical management. Thus, patients may not be as invested in answering the PROM compared to if it were administered by their treating clinician or in a trial context. Hence strategies to increase response have been built into the registry operations. An email delivered a full seven days after the original email reminding men to complete the EPIC-26 instrument was generally effective in increasing PROM returns across patient age, socioeconomic status, and day-of-the-week the reminder was sent. However, some differences in the magnitude of effect were observed with respect to patient age.

Email reminders are a simple and effective way of enhancing the response rate to patient reported outcome measures instruments [12, 13]. A study examined the effect of an automatic reminder email delivered within a month of the PROMs due date in men with prostate cancer enrolled in clinical trials [8]. This increased the response rate for the prostate cancer instrument from 75.7% to 79.7%. Given the programmed nature of the email reminder, the additional cost to the program administering the PROM is minimal compared to the large increase in returns. Furthermore, the automated prompt for men to self-report symptomatology may mean they answer in a more truthful way [14]. An additional benefit is that this approach decreases the error rate in collection; patients are entering data directly into the database system, rather than a member of the registry staff talking to a patient, interpreting what they have said, and then entering the data from a telephone conversation. Nevertheless, for those remaining non-responders, a telephone reminder at approximately 2 weeks after initial contact is part of the registry procedure. This contributes a small but measurable increase in the relative cumulative return rate, particularly for men aged under 65, and is consistent with prior studies [15, 16]. While it is suggested that phone reminders are not cost effective [17], the strategy of an initial reminder email reduces the amount of non-responders and consequently the amount of follow-up registry staff hours.

A limitation of this work is that our study population is older, exclusively male, and more likely to reside in socioeconomically advantaged areas. This is reflective of the risk profile for prostate cancer in our state [18] though it limits the generalisability of this research to other patient groups. Another limitation is that there was no control group, hence we have had to predict the effect of not receiving an automatic reminder. The precise reasons patients did not respond to the initial email were not explored in this study. It may be that the email was erroneously put into the patient’s spam folder, they were busy at the time of receipt, or they simply forgot about the email and the reminder email provided the impetus to complete the PROM. Patient specific factors such as amount of free time to answer such surveys were not measured with ancillary questions and these may partially explain the results observed. Further research to elucidate such factors may help improve the response rate to surveys in general.

In conclusion, an automated email reminder increased the response rate to a PROM instrument by almost 10% in a population-based cancer registry. We would encourage the use of this method as an effective and inexpensive strategy to increase the completion rate of email administered PROM instruments.

## Supplementary Information

**Additional file 1.** **Supplementary figures:** Study flow chart and cumulative percentage of EPIC-26 electronic completions by: day of the week, age, and socio-economic status.

## Data Availability

The datasets analysed during the current study are not publicly available as per the patient consent form.

## Abbreviations

PROM/PROMs: Patient reported outcome measures
EPIC: Expanded prostate cancer index composite
PCOR-Vic: Prostate cancer outcomes registry-Victoria
SES: Socio-economic status
