# Supplementary material for: Quantifying the effect email reminders have on patient reported outcome measure returns in a large prostate cancer registry
Source: J Patient Rep Outcomes. 2022 Mar 7;6:19. doi: 10.1186/s41687-022-00426-1 (PMC8901818; doi:10.1186/s41687-022-00426-1)

Supplementary figure 1. Study flow chart. N=5065 is the analytic sample.


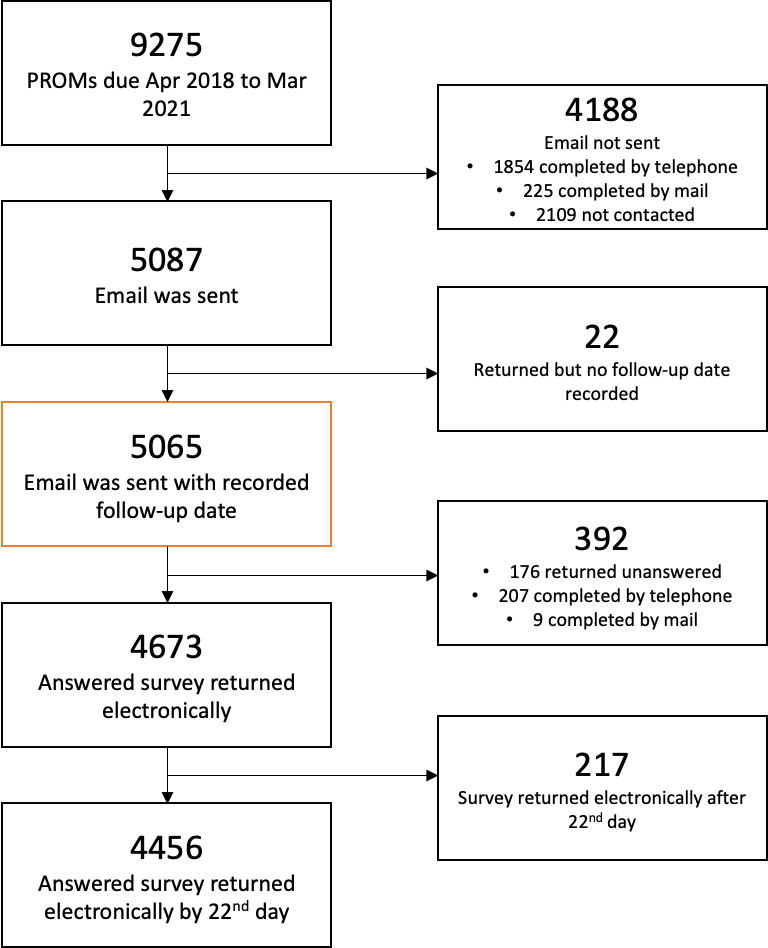


Supplementary figure 2. Cumulative percentage of EPIC-26 electronic completions by day since email sent. Red dotted line = email reminder sent following 8^th^ day. Lines plotted for all patients, by day of the week first email sent, age group and socioeconomic quartile.


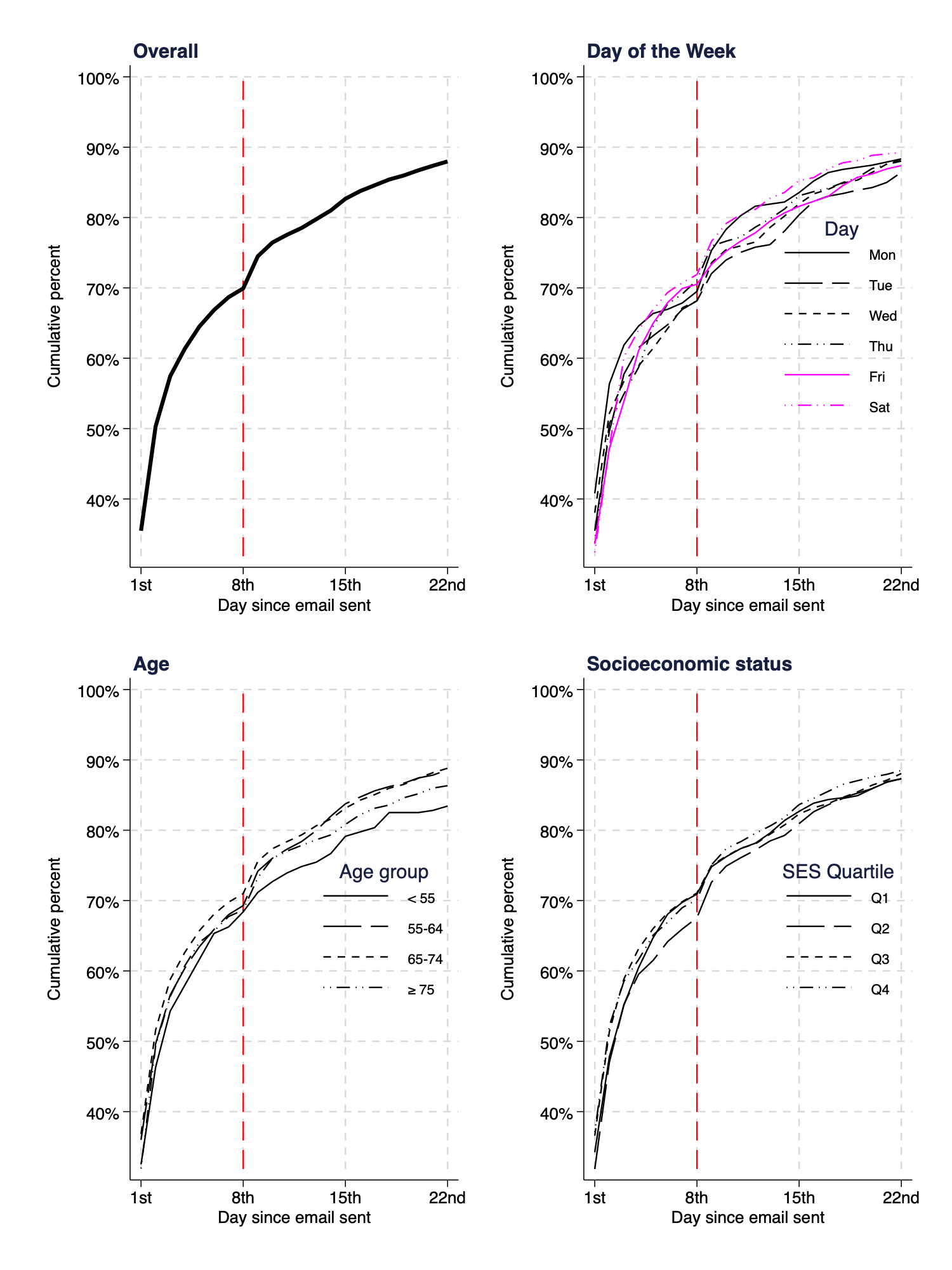

Supplement: Supplementary file 1 — Additional file 1. Supplementary figures: Study flow chart and cumulative percentage of EPIC-26 electronic completions by: day of the week, age, and socio-economic status. [file 41687_2022_426_MOESM1_ESM.docx]
